# Supplementary material for: Defining relictual biodiversity: Conservation units in speckled dace (Leuciscidae: Rhinichthys osculus) of the Greater Death Valley ecosystem
Source: Ecol Evol. 2020 Sep 1;10(19):10798–817. doi: 10.1002/ece3.6736 (PMC7548178; doi:10.1002/ece3.6736)
Supplement: Supplementary file 1 — Appendix S1 [file ECE3-10-10798-s001.docx]

**Appendix S1: Death Valley Subspecies**

Speckled dace within the Death Valley region exhibit high levels of diversity among the many isolated sites they inhabit. Population isolation has led to the proposal of five speckled dace subspecies within the Owens (N=2) and Amargosa (N=3) watersheds. All five subspecies are recognized by Nevada and California as being species of special concern (Moyle *et al.* 2015), and mitochondrial DNA data has supported the distinctiveness of each subspecies (Oakey, Douglas, & Douglas, 2004; Smith, Chow, Unmack, Markle, & Dowling, 2017).

Two subspecies are found in the Owens River Valley: Long Valley speckled dace (*R. o.* ssp 12), and Owens River speckled dace (*R. o.* ssp 2). Long Valley subspecies is the most imperiled freshwater fish in California (Moyle, Katz, & Quiñones, 2011). It is feared extirpated from the wild as of 2019. Its sole habitat is restricted to Whitmore Hot Springs – a thermal-spring complex that receives the partially chlorinated outflow of a public swimming pool (Moyle, Quiñones, Katz, & Weaver, 2015). Both morphological and genetic studies have confirmed its status as a distinct taxon, but to date it has not been formally described (Furiness, 2012; Oakey, Douglas, & Douglas, 2004; Sada, Britten, & Brussard, 1995).

The Owens River speckled dace also has a tenuous existence. The subspecies has been extirpated from eight of 17 historically known sites, and existing populations are only left in Fish Slough, Round Valley, and areas around Bishop, CA (Moyle, Quiñones, Katz, & Weaver, 2015). Habitat fragmentation has occurred due to export of water to Los Angeles following construction of aqueducts in 1913 and 1970 (Hollett, Danskin, McCaffrey, & Walti, 1991). This subspecies was once grouped with Ash Meadows speckled dace (Gilbert, 1893) until being recognized as a distinct population (Deacon & Williams, 1984; Williams, Hardy, & Deacon, 1982). Sada et al. (1995) also recognized morphological and genetic variation among Owens River populations, but did not determine additional splitting of the subspecies was warranted.

Three additional subspecies are found in the Amargosa Drainage: Ash Meadows speckled dace (*R. o. nevadensis*), Amargosa Canyon speckled dace (*R. o.* ssp 1), and Oasis Valley speckled dace (*R. o.* ssp 6). Ash Meadows speckled dace is the only species in the region afforded federal protection under the U.S. Endangered Species Act (ESA: Federal Register 1983). This subspecies is found within Ash Meadows National Wildlife Refuge, which was founded in 1984 to provide protection for all of the unique fauna endemic to Ash Meadows (Deacon & Williams, 1991). This taxon was initially described as a different species (*R. nevadensis*: Gilbert, 1893) before later being designated a subspecies of *R. osculus* (Hubbs, Miller, & Hubbs, 1974).

The upper Amargosa River is home to Oasis Valley speckled dace. It is currently restricted to Fleur de Lis Springs and a short portion of the upper Amargosa River near Beatty Nevada (Sada, Britten, & Brussard, 1995). Amargosa Canyon speckled dace is found in the lower Amargosa River, and has been evaluated to be in danger of extinction within the next 50 years (Moyle, Katz, & Quiñones, 2011). Today it is mostly confined to Willow Creek and Willow Creek Reservoir (Scoppettone, Hereford, Rissler, Johnson, & Salgado, 2011; Williams, Hardy, & Deacon, 1982). It has been extirpated from a spring north of Tecopa (Miller, 1938; Moyle, Quiñones, Katz, & Weaver, 2015). This subspecies was once grouped with *R. o. nevadensis* (Gilbert, 1893; La Rivers, 1962) and later recognized as distinct from the Ash Meadows subspecies (Deacon & Williams, 1984; Williams, Hardy, & Deacon, 1982).

**Appendix 1 References**

Deacon, J. E., & Williams, C. D. (1991). Ash Meadows and the legacy of the Devils Hole pupfish. In W. L. Minckley & J. E. Deacon (Eds.), *Battle Against Extinction: Native Fish Management in the American West* (pp. 69–87). Tucson, AZ: University of Arizona Press.

Deacon, J. E., & Williams, J. E. (1984). Annotated list of the fishes of Nevada. *Proceedings of the Biological Society of Washington*, *97*(1), 103–118.

Furiness, S. J. (2012). *Population Structure of Death Valley System Speckled Dace (*Rhinichthys osculus*)* (MS). Texas A&M University Corpus Christi, Corpus Christi, TX.

Gilbert, C. H. (1893). Report on the fishes of the Death Valley expedition collected in southern California and Nevada in 1891, with descriptions of new species. *North American Fauna*, *7*, 229–234. Retrieved from http://www.fwspubs.org/doi/pdf/10.3996/nafa.7.0003

Hollett, K. J., Danskin, W. R., McCaffrey, W. F., & Walti, C. L. (1991). *Geology and Water Resources of Owens Valley, California* (No. 2370). Washington, DC: US Government Printing Office.

Hubbs, C. L., Miller, R. R., & Hubbs, L. C. (1974). Hydrographic history and relict fishes of the north-central Great Basin. *Memoirs of the California Academy of Sciences*, *7*, 1–259.

La Rivers, I. (1962). *Fishes and Fisheries of Nevada*. Carson City, NV: Nevada Fish and Game Commission.

Miller, R. R. (1938). Record of the fresh-water minnow *Apocope nevadensis* from southeastern California. *Copeia*, *1938*(3), 147–147.

Moyle, P. B., Katz, J. V., & Quiñones, R. M. (2011). Rapid decline of California’s native inland fishes: a status assessment. *Biological Conservation*, *144*(10), 2414–2423.

Moyle, P., Quiñones, R. M., Katz, J., & Weaver, J. (2015). *Fish species of special concern in California third edition* [California Department of Fish and Wildlife Report]. Retrieved from California Department of Fish and Wildlife website: www.wildlife.ca.gov

Oakey, D. D., Douglas, M. E., & Douglas, M. R. (2004). Small fish in a large landscape: diversification of *Rhinichthys osculus* (Cyprinidae) in western North America. *Copeia*, *2004*(2), 207–221. doi: 10.1643/CG-02-264R1

Sada, D. W., Britten, H. B., & Brussard, P. F. (1995). Desert aquatic ecosystems and the genetic and morphological diversity of Death Valley system speckled dace. *American Fisheries Society Symposium*, *17*, 350–359.

Scoppettone, G. G., Hereford, M. E., Rissler, P. H., Johnson, D. M., & Salgado, A. (2011). *Relative abundance and distribution of fishes within an established Area of Critical Environmental Concern, of the Amargosa River Canyon and Willow Creek, Inyo and San Bernardino Counties, California* (U.S. Geological Survey Report No. 2331–1258; p. 32). doi: 10.3133/ofr20111161

Smith, G. R., Chow, J., Unmack, P. J., Markle, D. F., & Dowling, T. E. (2017). Evolution of the *Rhinichthys osculus* complex (Teleostei: Cyprinidae) in western North America. *Miscellaneous Publications Museum of Zoology University of Michigan*, *204*(2), 1–83.

Williams, C. D., Hardy, T. P., & Deacon, J. E. (1982). *Distribution and status of fishes of the Amargosa River Canyon, California* (p. 115). Sacramento, CA: Unpublished Report submitted to US Fish and WIldlife Service Endangered Species Office.
